# “I wish to remain HIV negative”: Pre-exposure prophylaxis adherence and persistence in transgender women and men who have sex with men in coastal Kenya

**DOI:** 10.1371/journal.pone.0244226

**Published:** 2021-01-19

**Authors:** Makobu Kimani, Elise M. van der Elst, Oscar Chirro, Elizabeth Wahome, Fauz Ibrahim, Nana Mukuria, Tobias F. Rinke de Wit, Susan M. Graham, Don Operario, Eduard J. Sanders

**Affiliations:** 1 KEMRI-Wellcome Trust Research Program, Kilifi, Kenya; 2 Department of Health, Kilifi County, Kilifi, Kenya; 3 Department of Global Health, Amsterdam Institute for Global Health and Development, University of Amsterdam, Amsterdam, Netherlands; 4 Department of Global Health, University of Washington, Seattle, Washington, United States of America; 5 Department of Public Health, Brown University, Providence, Rhode Island, United States of America; 6 Nufield Department of Medicine, Oxford University, Oxford, United Kingdom; Ohio State University, UNITED STATES

## Abstract

**Background:**

Transgender women (TGW) and men who have sex with men (MSM) in sub-Saharan Africa have high HIV acquisition risks and can benefit from daily pre-exposure prophylaxis (PrEP). We assessed PrEP adherence by measuring tenofovir-diphosphate (TFV-DP) levels and explore motives for PrEP persistence in TGW and MSM.

**Methods:**

Participants were enrolled in a one-year PrEP programme and made quarterly visits irrespective of whether they were still using PrEP. At their month 6 visit, participants provided a dried blood spot to test for TFV-DP levels; protective levels were defined as those compatible with ≥4 pills per week (700–1249 fmol/punch). Before TFV-DP levels were available, a sub-set of these participants were invited for an in-depth interview (IDI). Semi-structured IDI topic guides were used to explore motives to uptake, adhere to, and discontinue PrEP. IDI data were analyzed thematically.

**Results:**

Fifty-three participants (42 MSM and 11 TGW) were enrolled. At month 6, 11 (20.7%) participants (8 MSM and 3 TGW) were lost to follow up or stopped taking PrEP. Any TFV-DP was detected in 62.5% (5/8) of TGW vs. 14.7% of MSM (5/34, p = 0.01). Protective levels were detected in 37.5% of TGW (3/8), but not in any MSM. Nineteen IDI were conducted with 7 TGW and 9 MSM on PrEP, and 1 TGW and 2 MSM off PrEP. Unplanned or frequent risky sexual risk behaviour were the main motives for PrEP uptake. Among participants on PrEP, TGW had a more complete understanding of the benefits of PrEP. Inconsistent PrEP use was attributed to situational factors. Motives to discontinue PrEP included negative reactions from partners and stigmatizing healthcare services.

**Conclusion:**

While MSM evinced greater adherence challenges in this PrEP programme, almost 40% of TGW were protected by PrEP. Given high HIV incidences in TGW these findings hold promise for TGW PrEP programming in the region.

## Introduction

The World Health Organization (WHO) recommends pre-exposure prophylaxis (PrEP) to be offered to all populations at increased risk for HIV acquisition. Since May 2017, Kenya has been providing PrEP (daily pill; Tenofovir and Emtricitabine) through existing antiretroviral care programmes, family planning and partner notification services targeting adults meeting a wide range of risk criteria [1]. PrEP eligible participants include mostly young women in HIV high-burden locations [2], and HIV-negative partners from newly diagnosed index participants [3]. While men who have sex with men (MSM) are targeted for PrEP initiation in Kenya, PrEP uptake is limited. An assessment of missed opportunities for PrEP delivery among over 47533 MSM and 40338 young women in Kenya showed that MSM had substantially more (75% vs. 8%) missed opportunities to initiate PrEP than young women [4].

While PrEP efficacy has been proven also in sub Saharan Africa (SSA) [5], it is unknown what motivates the uptake and adherence to PrEP among MSM and transgender women (TGW) in Kenya. In a study in South Africa in 2015, among 167 MSM and TGW, approximately half of the participants started PrEP, but this study did not collect adherence data or laboratory markers of protection [6]. In a recent study in coastal Kenya among 170 MSM eligible for PrEP, 140 (82%) started it and 64 (58%) reported PrEP use at the end of study [7]. Among 76 MSM who reported PrEP use and had Tenofovir Diphosphate (TFV-DP) determined 6–12 months after PrEP initiation, only 11 (14.5%) had protective TFV-DP concentrations in blood plasma corresponding with ≥4 tablets a week [7]. To our knowledge, this is the only study that has reported PrEP protective levels in MSM in SSA, and no study has reported PrEP protective levels among TGW in SSA.

The low protective tenofovir concentrations in MSM in coastal Kenya contrast with results from a large demonstration study among MSM and TGW in the US that reported high PrEP protective levels (80%–82%) at follow-up visits over a similar duration [8]. These high protective tenofovir concentrations were attributed to favourable community perception and awareness of PrEP effectiveness [8].

Globally, TGW have a very high HIV-1 prevalence [9] compared to other at-risk groups, and have been largely overlooked in the HIV epidemic response in SSA [10]. Our recent work in Kenya demonstrated a high HIV-1 incidence (20.6 per 100 person years) among few TGW, indicative of an unmet PrEP need [11]. While both MSM and TGW were interested in starting PrEP, TGW expressed reservation to access PrEP at a public health facility, and indicated that they would prefer to collect PrEP from a community-based lesbian, gay, bisexual and transgender (LGBT) organization [11]. Earlier, we showed that PrEP adherence among MSM was impacted by a range of behavioural and psychosocial factors, including alcohol intake, travel, forgetfulness, and environmental barriers such as stigma and unfriendly healthcare providers [12].

We set out to assess PrEP adherence among a sample of MSM and TGW who participated in a PrEP program in coastal Kenya. We measured protective TFV-DP levels in blood samples and qualitatively explored motives for PrEP use and persistence in a sub-set of participants. Findings can inform development of a comprehensive and synergistic approach to improving PrEP adherence for MSM and TGW.

## Materials and methods

### Site

The study took place in a large Government hospital in coastal Kenya where PrEP services were provided along with HIV care services. Participants received PrEP services at a specialized office site for members of key populations, located adjacent to the general HIV care clinic. Site operations have been described previously [11]. In brief, MSM and TGW were offered free HIV counselling and testing; those testing HIV-1 positive were linked to care and offered ART. Participants testing HIV negative were offered PrEP provided free of charge by the Ministry of Health. Through a partnership with the community-based organization *AMKENI* supporting MSM and TGW members, participants were contacted to enroll in the PrEP cohort [11].

### PrEP-cohort

Between January and March 2018, HIV-negative MSM and TGW who had previously participated in a HIV incidence and PrEP interest study (completed mid 2017) were invited to enroll in the one-year PrEP cohort [11]. Participants were 18 years or older, and their HIV-negative status was confirmed at enrolment using a nationally recommended HIV testing algorithm [13]. Participants received 350 KSh (3.5 US$) to cover participation costs for each scheduled visit. PrEP initiation and maintenance followed national guidelines, including a one-month PrEP-supply (irrespective of creatinine result); a two-month supply for the next two months, and a quarterly supply thereafter. Participants were reminded of their upcoming clinic visit 24 hours before the visit date. Physical tracing was done for those who did not attend their assigned visit or who were unreachable on phone. At each visit, participants were provided with standardized PrEP adherence counselling, supporting participants to take PrEP at a regular moment in the day and discussing possible adherence challenges. Participants who had stopped taking PrEP were encouraged to re-start.

### PrEP-adherence

At scheduled visits participants were asked about their PrEP use, if they had missed pills, and how they would rate their adherence (form in S1 Questionnaire).

As most participants stated that their adherence was either good or excellent, we categorized participants as consistently adherent or inconsistently adherent if they reported that they had taken their last PrEP within 2 days or more than 2 days ago from their month 6 clinic visit.

### Dried Blood Spot sample collection and TFV-DP levels

At the 6-month visit, a Dried Blood Spot (DBS) sample to measure TFV-DP was collected from PrEP-taking participants, stored frozen (in minus 20°Cs), and shipped to a laboratory in Colorado, USA [14]. The concentration of TFV-DP in DBS was evaluated in fmol/punch and corresponded to the following weekly PrEP doses: below lower limit of quantification (LLOQ), LLOQ to 350 fmol/punch (< 2 tablets per week), 350 to 699 fmol/punch (2 to 3 tablets per week), and 700 to 1249 fmol/punch (4 to 6 tablets per week). Protective levels of TFV-DP were defined as ≥ 700 fmol/punch consistent with having taken ≥ 4 tablets a week [15, 16].

### In-depth interviews

Based on the 6-month visit, a purposefully selected sub-set of participants with consistent or inconsistent PrEP adherence (defined above), or who had stopped taking PrEP, was invited for an IDI. Participants received Ksh 500 (5 US$) to compensate for the IDI. IDI were conducted by the lead author (MK) who had previously conducted the PrEP interest and HIV-1 incidence assessment among MSM and TGW in the same community [11]. Summary notes of each interview were written shortly after the interview and discussed with a senior qualitative researcher (EvdE). All TGW in follow up and a convenient sample of MSM participants were interviewed. Details on consolidated criteria for reporting qualitative studies (COREQ) and the IDI topic guide for TGW are provided as S1 File.

### Data collection and analysis

Data cleaning, recoding and quantitative analysis was done using Stata 15.0 (Stata Corp LLC, College Station, Texas, USA). Descriptive statistics were used to compare baseline socio-demographic and behavioural characteristics of MSM and TGW. Associations between binary or categorical variables were investigated using Chi-square or Fisher’s exact tests.

IDIs focused on PrEP use and factors that influenced adherence. Interviews were semi-structured, and topics included PrEP knowledge, motivation for uptake, barriers and facilitators to adherence, and support needs. Specific prompts were included if the interviewee disclosed PrEP discontinuation or identified as TGW. Additional topics explored gender identity, HIV risk-taking perception, experience with public health services, and use of feminizing hormones (semi-structured interview guide in S1 File). IDIs were conducted in Kiswahili and audio-taped. IDIs were facilitated by a member of the research team (MK) and lasted approximately 45 minutes to 1 hour each.

Recordings were translated into English and transcribed verbatim. Data were managed using NVivo (version 11.4.1). Two qualitative researchers independently coded the transcripts and agreed on the final codebook used for the analysis. Braun and Clarke [17] thematic analysis was applied. A social ecological model, developed by Poundstone et al. [18], was used to organize factors (e.g., social and structural, environmental, individual practices) related to PrEP use. We triangulated quantitative findings on PrEP adherence and TFV-DP levels with qualitative perspectives on PrEP motives, experiences, and discontinuation.

This study was approved by the KEMRI scientific and ethical review unit (KEMRI/SERU/CGMR-C/0073/3418). All participants provided written informed consent prior to all study procedures.

## Results

In total, 53 participants (42 MSM and 11 TGW) were enrolled. MSM and TGW participants had similar age distributions with most (52.8%) between 25–34 years old. Two thirds (62.3%) reported only primary level education, the majority (83.0%) were single, and over half (52.8%) reported active sex work. Inconsistent condom use levels were similar in MSM and TGW. All TGW vs. 28.6% of MSM reported the receptive or versatile role during anal sex (Table 1).

**Table 1:** Characteristic of MSM and TGW PrEP cohort participants at enrolment, follow up status and PrEP use at month 6, coastal Kenya, 2018.

		MSM n = 42	%	TGW n = 11	%	Total n = 53	%	p-value
Characteristic								
**Age group**	18–24	12	28.6	3	27.3	15	28.3	0.60
	25–34	21	50.0	7	63.6	28	52.8	
	>34	9	21.4	1	9.1	10	21.4	
**Education**	Primary	28	66.7	5	45.4	33	62.3	0.35
	Secondary	11	26.2	4	36.4	15	28.3	
	Higher	3	7.1	2	18.2	5	9.4	
**Marital status**	Single	35	83.3	9	81.8	44	83.0	0.98
	Married	4	9.5	1	9.1	5	9.4	
	Separated/Divorced	3	7.1	1	9.1	4	7.6	
**Religion**	None	8	19.1	1	9.0	9	16.0	0.73
	Christian	16	38.1	5	45.0	21	39.6	
	Muslim	18	42.8	5	45.0	23	43.0	
**Sex work**	Yes	21	50.0	7	63.6	28	52.8	0.42
	No	21	50.0	4	34.4	25	47.2	
Anal sex role*	Insertive	28	66.7	0	0.0	28	52.8	**<0.01**
	Receptive	1	2.4	7	63.6	8	15.1	
	Both	11	26.2	4	36.4	15	28.3	
Condom use in past 3 months*	Always	13	31.0	4	36.4	17	32.1	0.55
	sometimes	22	52.4	7	63.6	29	54.7	
	Never	5	11.9	0	0.0	5	9.4	
**Follow up status at month 6**								
**In follow up and on PrEP**	Yes	34	81.0	8	72.7	42	79.3	0.55
	No	8	19.1	3	27.3	11	20.7	
**Adherence at Month 6 based on self-report**	Consistent^	17	50.0	6	75.0	23	54.8	0.20
	Inconsitent^	17	50.0	2	25.0	19	45.2	
**TFV-DP level**	Detectable	5	14.7	5	62.5	10	23.8	**<0.01**
	Undetectable	29	81.3	3	37.5	32	76.2	
**Corresponds to pills/week**	4–6 pills	0	0.0	3	37.5	3	7.1	**<0.01**
	2–3 pills	1	2.9	1	12.5	2	4.8	
	<2 pills	4	11.8	1	12.5	5	11.9	
	No pills	29	85.3	3	37.5	32	76.2	

*Two MSM did not provide a response.

^ Defined as consistently and inconsistently adherent if days between the last date PrEP was taken and the month 6 clinic visit date was < 3 days or ≥ 3 days, respectively.

TFV-DP-Tenofovir-diphosphate.

### TFV-DP levels

At month 6, 11 (20.7%) participants (8 MSM and 3 TGW) had stopped taking PrEP or were lost to follow up. Of the 42 participants still taking PrEP, any TFV-DP was detected in 62.5% (5/8) of TGW vs. 14.7% (5/34, p = 0.01) of MSM (Table 1). Overall, 3 TGW and no MSM had protective TFV-DP drug levels as per DBS analysis. Among the 53 individuals who initiated PrEP, only 6% (3/53) remained in PrEP care and had protective levels of adherence at 6 months.

### Qualitative findings

A total of 19 IDIs were conducted, including 11 MSM (2 consistently adherent; 7 inconsistently adherent; 2 stopped use) and 8 TGW (6 consistently adherent; 1 inconsistently adherent; 1 stopped use; see Table 2). IDI-participants with consistent PrEP use had a higher proportion of any TFV-DP than participants with inconsistent PrEP use (75.0% vs. 14.2%, p<0.02; data on days PrEP missed, days in a row missed and TFV-DP level in S1 Table). We identified four main domains: two key facilitators and two barriers to PrEP use and adherence. Table 3 outlines these main domains and presents sub-themes and representative quotes.

**Table 2:** Characteristics of in-depth interview MSM and TGW participants, PrEP adherence dried blood spot Tenofovir levels, coastal Kenya.

		MSM n = 11	%	TGW n = 8	%	Total n = 19	%	p-value
Characteristic								
**In follow up and on PrEP**	Yes	9	81.8	7	87.5	16	84.2	1.00
	No	2	18.2	1	12.5	3	15.8	
**Adherence at Month 6 based on self-report**	Consistent^	2	22.2	6	85.7	8	50.0	**0.04**
	Inconsitent^	7	77.8	1	14.3	8	50.0	
**TFV-DP level**~	Detectable	2	22.2	5	71.4	7	43.8	0.13
	Undetectable	7	77.8	2	28.6	9	56.3	
**Approximate pills/week**	4–6 pills	0	0	3	42.8	3	18.8	0.07
	2–3 pills	0	0	1	14.3	1	6.3	
	<2 pills	2	22.2	1	14.3	3	18.8	
	No pills	7	77.8	2	28.6	9	56.3	

^~^ TFV-DP -Tenofovir-diphosphate not known at the time of conducting interviews.

^ Defined as consistently and inconsistently adherent if days between the last date PrEP was taken and the month 6 clinic visit date was < 3 days or ≥ 3 days, respectively.

**Table 3:** Summary of themes, sub-themes, and representative quotes identified from in-depth interviews among MSM and TGW participants, coastal Kenya.

Major themes		Sub-Themes	Representative quote
***Motives to start PrEP***			
1	High HIV infection risk	Unplanned/frequent risky sexual behaviour	*Since I started taking up PrEP I feel a lot safer*. *I have the same number of partners but I have much more sex*, *I feel more energetic* (TGW 27 years, consistently adherent*)- IDI 011
			*I would say it* [who should take PrEP] *depends on the sex role*. *If they are bottoms*, *they should get it just like Trans*. *You know the anal region is weak and is not used to rough action so anyone that is getting anal sex should be considered*. *It’s not about MSM or Trans*. *It’s the risk (TGW*, *20 years*, consistently adherent*)- IDI 013
		Multiple sexual partners of unknown HIV status	*Sometimes I may not be able to convince all my partners to use a condom*. *(MSM*, *24 years*, consistently adherent*)- IDI 001
2	Gaining more sexual freedom	Risk compensation	*… before I knew about PrEP I had two partners*. *When I started using PrEP*, *I added two more [partners] as I felt protected [by PrEP]*. *Now I have four partners*. *(MSM*, *31 years*, inconsistently adherent*)- IDI 012
		Being in difficult situations	*Sometimes I may not be able to convince all my partners to use a condom*. *Sometimes someone also gets bored of condoms but is still afraid of infections that is where PrEP comes in to help*. *(TGW*, *39 years*, *stopped PrEP)- IDI 017*
3	To convenience condomless sex	Monetary gain	*… the other problem is that if you insist on condom use then the money they [clients] are giving you becomes less… (TGW*, *24 years*, inconsistently adherent*)-IDI 002
***PrEP adherence facilitators***			
1	Integrated healthcare services	Assisted disclosure on PrEP use	*We had to come to the clinic together* [with partner], *and the counsellor explained to both of us what PrEP was and then finally he [my partner] understood (TGW*, *27 years*, consistently adherent*)- IDI 010
		Disclosure of PrEP use	*My immediate family know*, *…I live at home and we really can’t have any secrets*. *So*, *they have seen my [PrEP] pills… …they also remind me when it is time to take my pills*. *(TGW*, *21 years*, consistently adherent*) -IDI 013
		Hope for future improved PrEP delivery models	*…PrEP that lasts longer… I would want a pill that can be taken like once a month*, *or even better*, *make an injection*. *This takes away all the stigma*, *carrying [PrEP] pills around*. *(TGW*, *22 years*, consistently adherent*)-IDI 014
***Reasons for inconsistent PrEP use***			
1	Current PrEP formulation	Struggle with daily dosing	*… I think it is a nuisance when it comes to the daily dosing*. *Having to remember when to take PrEP*, *having to interrupt my fun just to take a drug*. *That is the nuisance… (TGW*, *28 years*, inconsistently adherent*)- IDI 005
2	External influence and circumstances	HIV related stigma	*…there is one person who saw me with them [PrEP pills] and asked me whom they belonged to*. *I told him that they were mine and he told me that I am a gone case… ARVs look just like this… He went spreading the news*. *(MSM*, *21 years*, *stopped using PrEP)-IDI 018*
		Concomitant drug use	*…later that same evening I went out drinking with my friends*, *and I used some drugs [Khat*?*]*. *The drugs [combination of PrEP and khat] made me get confused,…. I used another pill*. *I got very bad diarrhea and stopped PrEP for a while… (TGM*, *22 years*, consistently adherent*)-IDI 014
3	Uncertainty about PrEP use	Fear of using drugs close to expiry	*I travelled and left them (PrEP pills] here*, *when I came back I found they were almost expiring*. *When I see that something is one month to expiry*, *I am afraid*. *(MSM*, *23 years*, *stopped PrEP)- IDI 018*
		Uncertainty about refill date	*…time moves so fast*. *Like now*, *I was called and told that I was late for my appointment*. *I was very surprised since I still had a lot of drugs*. *I thought that drugs are supposed to end that I come to the clinic*. *… (TGW*, *30 years*, consistently adherent*)-IDI 004
***Motives to discontinue PrEP***			
1	Undesirable dispensing venue	Move away from Government facility	*It* [PrEP] *should be private and specifically for us* (MSM). *…it should not be under Government*. *You know the problems we have with the Government… (MSM*, *31 years*, inconsistently adherent*)-IDI 003
2	Perceived stigma	Discrimination by healthcare providers	*We [TGW] may not be very comfortable at the Government facility as the staff there are too curious*. *They just ask questions for the sake of getting things to talk about not because it is related to our needs… No*, *I don’t think TGW would want to get services at the Government facility*. *(TGW*, *27 years*, consistently adherent*)- IDI 002

*Interviews done before PrEP drug levels were known. Defined as consistently and inconsistently adherent if days between the last date PrEP was taken and the month 6 clinic visit date was < 3 days or ≥ 3 days, respectively.

#### PrEP initiation motives

The most important driver of PrEP uptake was self-perceived high risk for HIV infection. For example, one TGW participant stated, “I wish to remain HIV negative. I know that being a trans is putting me at risk for HIV. So, when I heard that PrEP was available here [hospital], I was among the first to ask for it”. *(TGW*, *30 years*, *consistently adherent*, *IDI 004)*. Additional motives for PrEP uptake included the ability to exercise sexual freedom and protection in the context of sex work.

#### PrEP adherence facilitators

A key facilitator to PrEP adherence was access to an MSM- and TGW-friendly PrEP service site, staffed by competent providers. Participants appreciated that providers offered counseling to support use of PrEP and assistance in disclosure to partners and family members. TGW participants noted that being on PrEP had a positive effect on their overall outlook of life. For example, one stated:

*“*…*PrEP has been very useful to me*. *It has made me feel more alive and happier about my life*. *I even have better appetite and have more drive and purpose in my life*…*”*

*(TGW*, *24 years*, *consistently adherent*, *IDI 002*).

Taking feminizing hormone therapy (FHT) together with PrEP was not reported but appeared to be an issue for some TGW participants, one stated:

*“I do not have knowledge of* [FHT] *where to start*. *I would really need help in that area*. *I especially would like to get help in getting breasts*. *Sometimes you are with a client and he wants you to be more female*. *I would want help in that area*. *I do not have any worry about PrEP interacting with hormones*. *I would be OK…*.*”*

(*TGW, 24 years, consistently adherent, IDI 002*)

#### Inconsistent PrEP use

Struggling with daily dosing was mentioned as a challenge to PrEP adherence. Participants felt that daily PrEP was a nuisance and expressed desire to have long-acting formulations. Related to this was concomitant drug usage. One MSM narrated how taking alcohol and recreational drug use resulted in him having to temporarily stop taking PrEP:

… *I went out drinking and used some drugs [Khat]. The drugs [combination of PrEP and khat] made me get confused,…. I got very bad diarrhoea and stopped PrEP for a while*…

*(MSM, 24 years, inconsistently adherent, IDI 002)*.

When asked how participants could improve the PrEP program when given the opportunity, one TGW remarked:

I would create a PrEP that lasts longer. I would want a pill that can be taken like once a month or even better make an injection. This takes away all the stigma of need to carry pills around

*(TGW*, *22 years*, *consistently adherent*, *IDI 014*).

Another TGW participant who was among the three participants who had evidence of consistent PrEP use stated:

If it was possible I would like to have an injectable PrEP. Sometimes we have nowhere to keep our medications and an injection would be perfect. It is much easier to use and it is impossible to forget it

*(TGW*, *20 years*, *consistently adherent*, *IDI 013*)

Of note, these views on long-acting PrEP or injectable PrEP were spontaneously revealed by participants.

One of the main reasons for poor adherence noted amongst TGW was non-disclosure of PrEP use. The need to hide medication and the lack of an opportune moment to take it in the presence of others impacted adherence.

…*I have hidden them [PrEP pills], so no one knows about them, so if I forget to take them, there won’t be anyone to remind me*…

*(TGW, 28 years, inconsistently adherent, IDI 011)*.

#### Motives to discontinue PrEP

A key motive for PrEP discontinuation was concern about the government-affiliated nature of the PrEP clinic, as one MSM participant summarized “*It should not be under Government*. *You know the problems we have with the Government*.*”* Others noted discomfort with government-employed health providers, revealed by a TGW participant who commented “*…the staff there are too curious*. *They just ask questions for the sake of getting things to talk about*, *not because it is related to our needs… No*, *I don’t think TGW would want to get services at the Government facility*.*”* PrEP discontinuation also emerged out of pressure from intimate partners who demanded that participants stop using PrEP. A TGW in a heterosexual union narrated:

…*my partner found out about my using [PrEP] medications every day. I tried to explain to her… However, she does not know about me being with men, so she could not understand…. And demanded me to stop taking the [PrEP] drugs*…

*(TGW*, *39 years*, *stopped*, *IDI 018*).

## Discussion

This study provides quantitative and qualitative insights into PrEP adherence among MSM and TGW in coastal Kenya. Of the 42 participants who remained on PrEP 6 months post-initiation, 10 (23.8%) had any detectable drug levels, and only 3 participants (37.5%, or 3 of 8 TGW) had evidenced protective drug concentrations in dried blood spot samples. Among the 53 individuals who initiated PrEP, only 6% (3/53) remained in PrEP care and had protective levels of adherence at 6 months. There are limited data on PrEP uptake, adherence, and HIV-1 incidence reduction following programmatic PrEP uptake by any key population in sub-Saharan Africa, and no data on PrEP adherence among African TGW to our knowledge.

In a recent study in Mtwapa, Kenya, in which 14.5% of MSM participants had protective TFV-DP concentrations in dried blood spot samples corresponding with ≥4 tablets a week, HIV-1 incidence (3.6 per 100 person years) estimated among PrEP taking MSM was not different from the HIV-incidence (3.5 per 100 person years) among non-PrEP taking MSM [7]. In the latter study, four out of five MSM who acquired HIV-1 while reporting PrEP use had not taken it [7]. In another Kenyan study, among 347 18–24 year old women who were monitored for PrEP adherence at 6 months post PrEP initiation, only 15% took an average of 5 pills a week, suggesting limited prevention-effective adherence [19]. Findings from the current study are therefore consistent with previous research reporting poor adherence to daily PrEP among Kenyan populations.

Our findings contrast with a demonstration study of daily PrEP in the US showing high (>80%) PrEP protective levels among MSM and TGW participants [8], and an on-demand PrEP study in Europe showing that 71% of participants had tenofovir detected suggestive of last drug intake during the previous week [20]. The two Kenyan studies to date reveal that overall PrEP persistence was very low in MSM. In the present study, few MSM and TGW participants (5/53, or 9.4%) had detectable TFV-DP levels, indicating less than 4 pills taken during the week of assessment.

All study participants in this study were advised to use PrEP daily, conforming to Kenya guidelines. Our study was completed before event-driven PrEP use has been recommended for MSM by the World Health Organization in 2019 [21]. During IDI we did not explore if participants were using non-daily or event-driven approaches to PrEP use. Given the low PrEP persistence in two Kenyan studies, further studies might explore if an event-driven PrEP regimen is attractive to Kenyan MSM [7].

In a PrEP demonstration study among 376 participants in Amsterdam, event-driven PrEP was preferred by 27% of participants, who reported a lower number of sexual partners, total sex acts, and condomless sex acts with casual partners than participants taking daily PrEP [22]. In a recent educational intervention study providing “2-1-1” dosing information to over 3000 MSM in San Francisco, 23% opted for event-driven PrEP [23], and event-driven PrEP reduced medication use three-fold while preserving high rates of effective use [23]. Research on preferences, motivations, and user characteristics related to event-driven PrEP among MSM is Kenya is needed to inform HIV prevention programming.

HIV-1 incidence among TGW in sub-Saharan Africa is probably several fold higher than among MSM. Our initial estimate of 20.6 per 100 person years in a small cohort study of TGW in coastal Kenya [11] is now supported by incidence estimates among TGW in Nigeria (23.8 per 100 person years) [24] and in South Africa (31.0 per 100 person years) [6]. As protective drug concentrations among TGW taking PrEP were significantly higher than among MSM taking PrEP participants, our findings hold promise for PrEP programmes targeting TGW in the region.

Through qualitative interviews, we found that adherence was stronger among participants who described clear motivations for self-preservation and protection against HIV based on their acknowledgment of personal risk. In particular, TGW expressed strong desires to remain HIV negative and had better adherence indicators compared to MSM. MSM participants seemed to be less cognizant of their risk for HIV acquisition, corresponding with previous research on low perceived personal HIV risk as a reason for lowered PrEP adherence [25]. The reasons for stronger PrEP motives among TGW in this sample are unclear, but this may be due to stronger connections in the local TGW community and the presence of public health messaging linking HIV risk with receptive anal sex and TGW populations globally. In contrast to MSM, TGW associated the use of PrEP with a general better quality of life, and some TGW expressed interest to learn about PrEP and FHT. FHT could help to affirm their female gender identity, a finding corresponding with previous research on the synergistic benefits of PrEP and gender-affirming health services for TGW [26, 27].

Most respondents, irrespective of adherence level, expressed negative sentiments about accessing PrEP from a government-affiliated public health facility. Health care providers at government facilities were thought to be less prepared and competent to serve MSM and TGW, compared with trained staff at the research clinic. This corresponds with previous studies that documented some providers’ unease delivering PrEP to MSM and TGW patients [28] and other at-risk individuals due to personal beliefs [29]. By contrast, research has also shown transgender-sensitive and competent health care providers can increase TGW patients’ empowerment and desires to seek healthcare services [27]. In addition, alternative PrEP dispensing venues in Kenya including the use of community pharmacies can potentially expand the reach of PrEP to individuals and communities with discomfort seeking services at government-run facilities. PrEP-taking individuals may also benefit from a peer support model which has recently been shown to improve ART adherence among MSM in a pilot study in coastal Kenya [30, 31].

Participants voiced strong support for long-acting PrEP as a possible improvement for adherence. This view emerged spontaneously when opinions for improvement of the PrEP program were solicited. TGW stated that HIV-related stigma attached to the daily route of PrEP use was a barrier to adherence. HIV-related stigma has been previously described in studies with MSM who described conflicts between the protective effects of PrEP coupled with moral judgements about PrEP use from colleagues and general society [32]. Long-acting PrEP formulations may be a solution around this dilemma, as the HPTN 083 trial showed that long acting injectable PrEP among MSM and TGW was safe and reduced the estimated HIV incidence by 66% when compared to daily PrEP [33].

Our findings suggest a possible relationship between initial motives for PrEP uptake and actual adherence. Consequently, future PrEP programs may consider assessing PrEP motives during initial screening and intake in order to incorporate user-specific motives into personalized adherence counseling and interventions to support PrEP persistence. This information may also help identify users at risk for PrEP discontinuation—for example, individuals who express discomfort with clinic facilities or providers or concerns about partner or family disclosure.

Limitations to this research must be acknowledged. First, participants were enrolled in a clinic that specifically provides services to key populations, which might have influenced participants’ responses which favoured targeted services for key populations. This type of clinic is also relatively unique in the context of sub-Saharan Africa, thereby limiting generalizability. Second, the drug level outcomes as reported here were not available at the time of conducting the IDIs, and as a result could not be incorporated into interview protocols. Participants were informed of study findings in a generalized dissemination meeting, but not of individual drug level results. Third, while drug level assessments by DBS were informative, they were only able to reveal whether drugs were taken in the preceding 3–7 days and may not indicate PrEP adherence during longer periods [34]. Finally, due to the small sample size, findings might have limited generalizability.

## Conclusions

In summary, this paper provides evidence concerning the challenges to PrEP adherence and insights into the motives related to PrEP uptake and persistence among Kenyan MSM and TGW. The observed overall low levels of biologically protective PrEP concentrations in blood plasma in this sample were particularly alarming. However, relatively higher protective levels among TGW participants relative to MSM hold promise for PrEP programming for TGW in the region. Qualitative assessments suggested potential links between PrEP uptake motivation and actual adherence and call for PrEP services that are sensitive and supportive of MSM and TGW needs, concerns, and motivations.

## Supporting information

S1 Dataset (TXT)

S1 File (DOCX)

S2 File Consolidated criteria for reporting qualitative studies (COREQ): 32-item checklist. (DOCX)

S1 Questionnaire PrEP information, motivation and adherence. (DOCX)

S1 Table Summary of self-reported PrEP adherence among 16 MSM and TGW participants, coastal Kenya. (DOCX)
