# Supplementary material for: “I wish to remain HIV negative”: Pre-exposure prophylaxis adherence and persistence in transgender women and men who have sex with men in coastal Kenya
Source: PLoS One. 2021 Jan 19;16(1):e0244226. doi: 10.1371/journal.pone.0244226 (PMC7815127; doi:10.1371/journal.pone.0244226)
Supplement: S1 File — (DOCX) [file pone.0244226.s002.docx]

| Barriers and Facilitators to PrEP uptake and adherence among Men who have sex with men and Transgender Women at Malindi Sub-County Hospital  **IDI guide** |
| --- |

Interview group: **Participant Initiated on PrEP, but with poor adherence + being TGW participant**

Interviewer Initials: ______________

Date: __________________________

Start time ______________________

End time: _______________________

**Background information**

Study number: _________________

Visit code: _____________________

Age: ____

Enrolment date: _________________

Date initiated on PrEP: ____________

Adherence history: ___________________________________________________________

Risk assessment: _____________________________________________________________

**General Introduction**:

Thank you for agreeing to be part of this interview as part of your participation in the ongoing Malindi Tuamuke Tujikinge study. We would like to learn about how to better provide Pre-Exposure Prophylaxis (PrEP) services to participants in follow up at KEMRI. We would like to know how to better support men who have sex with men (MSM) and transgender women (TGW) to take PrEP and maintain good adherence over time.

I expect that this interview will last about 1 hour. I also want to remind you that you can always skip answering a question and you can end the interview at any time.

With your permission, we would like to tape record the interview for later transcription and translation to English for analysis. All information will be kept strictly confidential and as respondent, you will never be identified with any comment you make.

**Introduction to Interviews:**

We seek to explore the challenges influencing uptake and adherence to PrEP as well as the facilitators among MSM and TGW. HIV disproportionately affects key populations and TGW even more. PrEP, when used in combination with other prevention methods, significantly reduces the risk for HIV acquisition. Through this interview, we would like to better understand the difficulties MSM and TGW face to initiate PrEP, maintain good adherence to PrEP, identify reasons for discontinuation and factors that may improve adherence. Learning from your experiences will help address some of the challenges MSM and TGW face in the context of PrEP services, eventually improving future PrEP uptake and adherence. We hope to learn from you the best model to scale up PrEP access to key populations including TGW.

Do you have any concerns or questions? I am happy to address them before we start.

Now I’m going to turn on the recorder and we’ll get started …

**Ice-breaker**

Focus on interviewee’s psychological / physical well-being, and daily issues:

- How are you feeling today? How have you been*?*
  - *(make interviewee comfortable talking about his/her experiences)*
- For how long have you been a participant in this study?
- Please tell me a little about your gender identity
  - *Probe for TGW or GNC*
  - *Probe if sometimes identity swings back to male (MSM) and any effect this has on her personality and observance to instruction, laws, societal norms etc.*
  - *Probe into process leading to discovery on above identity, coming out process, associated stigma from family, school employers, health care providers*
  - *Probe for coping and support base including other TGW or GNC*
- Why do you think being transgender, has any association with higher risk for HIV acquisition, compared to MSM?
  - *Probe for issues on homelessness, lack of legal identification documents, difficulty getting and sustaining employment*
  - *Probe for (if appropriate) perceived or known sex drive differentials between TGW, and other MSM*
  - *Follow above probe with any known preferred sex practices peculiar to TGW compared to other MSM and reasons for that e.g. more unprotected anal receptive sex or condomless oral sex*

**Topic 1**. **General knowledge of PrEP**

- What do you know about PrEP?
- *Probe for:*
  - *Prevention vs treatment*
  - *How does it work?*
  - *PrEP’s usefulness (HIV risk reduction)*
  - *PrEP’s safety and effectivity (duration of protection /one pill, once a day)*
  - *PrEP’s target group (do you know who are eligible to take PrEP vs who are NOT eligible to take PrEP, why Is PrEP recommended for these groups)*

*If not mentioned – would you say PrEP is for TGW and why?*

- - *Why is there need for screening before starting PrEP*
  - *PrEP adherence (how often; can one take a break? What is required if you need to take a break)*
  - *What if occasional dose missed? How long is PrEP used for, and when should one stop?*
  - *Difference between PEP/PrEP*
  - *What is your opinion on combining condom use and PrEP?*
  - *Myths and Misconceptions about PrEP?*
  - *Probe for sources of any wrong/ incomplete information on PrEP, any impact such information has had on adherence and retention*

**Topic 2.** **Experience using PrEP**

- Please can you tell me when you started taking PrEP?
- What was your motivation to start PrEP? Do you think TGW are at higher risk for HIV acquisition? I was thinking of having a probe here on TGW identity as a motivator to PrEP uptake

*Probe for: sexual risk taking, condomless sex, transactional sex, group sex, coerced sex*

*Probe for: New relationships? Perceived self to be at high risk? Health worker’s recommendation? Peer pressure? Unknown HIV status of partner? Being a TGW?*

- Would you please describe your experience being on PrEP?
  - *(probe for regular doctor visits/medical screening; concerns regarding disclosure; concerns from significant others; stigma; fear of PrEP safety; misconceptions, other? Concerns of being outed as a TGW?)*
- Did you experience any side effects? How did you cope with them?
  - *Call the clinic? Came back to the clinic? Consulted a friend?*
  - *Did you feel supported during this time? How differently would you say you should have been supported? Is there a different way you feel TGW should be supported?*
  - How helpful would it be if KEMRI had a helpline for support? What kind of support would you expect?
- Do you know of any other place where PrEP is being offered?
  - *If public health care facility is mentioned, probe for desire/aversion to access PrEP there*
  - *Probe if the aversion based on personal experience at healthcare facilities or other TGW experiences*
  - *Probe if the negative experience is from healthcare provider or other people seeking health care services*
  - *Does the aversion to public health facilities only apply to preventative/elective services or would the respondent be hesitant to seek routine or even emergency health services?*
- What worries do you have in taking PrEP?
  - *(Probe: protection of own health; protection of partners’ health; concerns such as acquiring STIs; frequency risky (condomless) sex; fear for medical? /routine* tests; side effects; fear for stigma/discrimination; practicalities; *PrEP-hormones interactions? Regular visits to the clinic following a past negative healthcare* experience as a TGW? other?)
- How would you say your life has changed while taking PrEP?

*(Probe for: sexual behaviour; disclosure of PrEP use; relationship with partner(s); social support; perceived stigma; mental health status; gender-based violence; stopped taking hormonal therapies?)*

- - *Did sexual risk-taking increase once on PrEP? Or reduce? Probe for reasons/motivation behind either behavioral change*
- Think about the days that you missed taking a pill. What could have contributed to that?
- At times that you were taking PrEP every day, what was helping you to do that?
  - *(Alarm reminders, treatment supporters)*
- Sometimes people taking PrEP opt not to take it on certain days if they do not feel that at that particular time they are at risk or will not be doing anything putting them at risk for HIV acquisition. Do you sometimes do that?
  - *Probe for a clear understanding of reasons of risk*
  - *Probe for what the exact circumstances are*
- If the above breaks are in consultation with your healthcare provider, they are called ‘planned drug holidays.’ What is your opinion on planned drug holidays?
  - *Probe for when these breaks would be taken*
  - *Probe for how frequently in a year the participant feels breaks can be taken and for how long*
  - *Would a planned drug holiday enhance adherence and retention?*

**Topic 3**. **Personal and social stigma around PrEP**

- Have you experienced any stigma with regards to using PrEP?

*Probe for stigma at the health facility specific to transgender identity*

*probe also for imaginary fear and internal stigma, HIV related stigma from social networks?*

*Outed as a TGW in the event respondent is living a double life?).*

- To whom have you disclosed to that you are using PrEP? How many other people have you told that you are taking PrEP?
  - *(Probe: why this choice? What is your relationship with these people? Why is the disclosure important to you? What support was received or expected?)*
- Do you think there are people who suspect you are taking PrEP? Why?
  - If yes, how do you feel about it?
- Do you have worries about how other people will react if they learn you are on PrEP?
  - Why? Fears? Stigma? tell me more about that
- What problems have you encounter while using PrEP?
  - *(Probe for fears (reaction; rejection; suspicion; threats; partner reaction/break-up; HIV association/stigma); rumours in the community; discrimination of community members/family; harassment; misconceptions of others; practicalities, such as storage, frequent travels, using PrEP while on hormonal care, etc.)*
- Do you have other problems not directly related to the drug that influence your adherence?
  - *(Probe for mental health issues, alcohol use, drug abuse; time spent at the clinic; homelessness; regular violence from the public; past experience with healthcare) incarceration due to arrest for sex work,*

**Topic 4. Barriers/Facilitators to PrEP use:**

- What has been the most challenging for you taking PrEP?
  - (for TGW probe about integration of PrEP with hormonal care)
  - What do you think could be helpful in overcoming these challenges?
  - What kind of support have you received or expect?
- From your experience on PrEP, what sort of support would you say is helpful to give people on PrEP?

*Probe for need for trans specific healthcare facilities and point people at the facility*

- Have you been thinking of what can be helpful to improve your adherence?
  - *(probe for previous ways of coping with meds – setting of alarm for reminders, treatment buddy, changes in routines, preferred interval of refill visits etc.)*
  - *Revisit planned drug holidays if appropriate to do so or if respondent mentions it. Probe for actual planning of a drug holiday, is there a need for follow up when holiday is almost coming to an end*
- As an individual what would you say could help you to better adhere to PrEP?

(probe for: support group? On-going counselling? Couple counselling? Social media support? *LGBT organization support? setting of alarm for reminders, treatment buddy, changes in routines, trans competent providers, etc*

- From your experience on PrEP, what sort of support would you say is helpful to give MSM and TGW on PrEP?
- How do you think you can be better supported by KEMRI in taking and adhering to PrEP?
  - *(Probe for professional healthcare and social; trans friendly providers, community; PrEP support groups facilitated by KEMRI)*
- What role can your partner (and / or family, friend) play in helping you adhere to PrEP
  - *Probe for any worried about intimate partner violence, social harms linked to PrEP use*
  - *Does coming to/being seen at the coming to the key populations clinic portend and social harm or even possible IPV*
- What role can a HCW can play to mitigate any of the above harms?
- How can we help you staying on PrEP?

**Topic 5.** **Barriers/Facilitators to PrEP provision at KEMRI Clinic**:

- How was the enrolment process to PrEP?
  - *Probe for emotional preparedness, counselling support, dissemination of information, baseline screening tests, waiting time*
- What do you like about how KEMRI introduced PrEP to you?
- What do you dislike about how KEMRI introduced PrEP to you?
  - *(Probe about counselling and clinical services, screening tests,*
- Do you have any suggestions to change?
- Please share with me any specific complaints you may have (need to be addressed or myths and/or misinformation you would like clarified)

**Topic 6.** **Accessibility of PrEP**:

- Has there been a situation that you ran out of PrEP? Can you describe?
  - What kind of support did you receive?
  - What role do you think KEMRI could have played?
- Apart from KEMRI where else do you consider getting a refill from if your medications ran out?
  - Have you ever encountered any problems?
  - *Probe for role of trans lead community based organizations*
  - How did you get them? Who picked them up?
  - How different would you say you can be supported with regards to refills?
- How would you prefer the interval of the refill appointments to be?
  - Why do you think that this choice will help you take your medication correctly?
  - If more than one-month refill date probe for HIV screening eg Use of OST for screening at home
  - *Probe for knowledge and desire for longer acting PrEP*
- How would you prefer KEMRI to provide the services?
  - Probe on engaging with a clinician, counsellor, pharm tech or one service provider?
- Based on your experience on PrEP how do you think it can be made easily accessible to MSM and TGW?
  - Partnerships with LGBT organization, peers, pharmacies, trans targeted PrEP campaigns,

**Topic 7. Recommendations and way forward**

- If you were in charge of a PrEP program for TGW, what would you desire to see done differently?
  - *Probe for venue, drug delivery route, publicity about PrEP, outreach activities etc.*
  - *Probe on desired sources of information*
  - *Probe on desired skills for health care providers*
  - *Probe on desired risk reduction strategies specific to TGW*
- What additional services do you think are important to integrate into a prevention program
  - *Probe for mental health services, screening for and referral for depression, harmful alcohol use and drug abuse*
- *What do you think of Transgender support groups? (online, or membership of TGW CBO?)*
- *Support to unite with and educate family and allies of TGW?(family acceptance of TGW)*
- *Special counselling services*
- *Crisis TGW cell (HOT line) to offer support and assistance to address violence / harassment and discrimination)*
- *More resources / information about transgender health, advocacy, and human rights*
- Any other issues that you would like to add that I may have missed?

*Thank you so much for your time and for answering the questions. We have come to the end of this interview, before we end, is there anything you think I should know? Do you have any question for me?*

*END*
