# Supplementary material for: “I wish to remain HIV negative”: Pre-exposure prophylaxis adherence and persistence in transgender women and men who have sex with men in coastal Kenya
Source: PLoS One. 2021 Jan 19;16(1):e0244226. doi: 10.1371/journal.pone.0244226 (PMC7815127; doi:10.1371/journal.pone.0244226)
Supplement: S1 Questionnaire — (DOCX) [file pone.0244226.s004.docx]

**S1 Questionnaire: PrEP Information, Motivation and Adherence**

Q1. Participant ID __ __ __ __ __ __

Q2. Study Date __ __ / __ __ / __ __ __ __ dd – mm - yyyy

SECTION A: PrEP Adherence

PB1. When was the last date you took your PrEP? __ __ - __ __ - __ __ __ __ dd - mm - yyyy

PB2. When was the last time you took your PrEP?(Time in 24hr) __ __ : __ __ hh : mm

PB3. Sometimes people have difficulty remembering or taking their medicines every day. During the last month, did you miss taking your PrEP on one day or more?

1 Yes

0 No Skip to PB6

PB4. How many days did you miss taking your PrEP? __ __ Days

97 Don't Know

98 Refuse to Answer

PB5. What was the most days in a row that you did not take PrEP? __ __ Days

97 Don't Know

98 Refuse to Answer

PB6. In the last 30 days, how good a job did you do at taking your medications in the way you were supposed to? (Choose one)

0 Very poor

1 Poor

2 Fair

3 Good

4 Very Good

5 Excellent

PB7. In the last 30 days, how often did you take your PrEP in the way you were supposed to? (Choose one)

1 Never

2 Rarely

3 Sometimes

4 Usually

5 Almost always

PB8. *Put a mark on the line below at the point showing your best estimate of how much medication you have taken in the last month. We would be surprised if this was 100% for most people.*

Examples:

0% means you have taken no medication

50% means you have taken half of your medication

100% means you have taken every single dose.

Visual analogue scale value

__ __ __

PB9. In general, how often do you take your PrEP**?** 0 None of the time

(Choose one) 1 A little of the time

2 Some of the time

3 A good bit of the time

4 Most of the time

5 All of the time

PB10. During the past month, what has helped you to remember to take your pills?

(Check all that apply)

__ Nothing

__ Alarm

__ Family

__ Friends

__ My partner, who is taking ART

__ Associate it with daily activity

__ Watch/clock

__ Pill carrier for 1-2 doses

__ Radio

__ Cellphone

__ Other, specify: _______________________________________

PB11. During the past month, what caused you not to take your pills?

(Check all that apply)

__ Nothing (I did not miss any pills)

__ Busy

__ Forgot

__ Traveled or other change in routine

__ Missed a refill/ran out

__ Illness

__ Lost or stolen pills

__ Shared pills with someone else

__ Alcohol use

__ Side effects/drug is toxic or harmful

__ Too many pills

__ Felt depressed/overwhelmed

__ Confidentiality/stigma

__ Other, specify: ____________________________________________________

PB12. For a variety of reasons, other persons may want to use your PrEP medication. Do you think that someone other than you has used any of your tablets?

1 Yes

0 No Skip to PB15

PB13. Who do you think has used any of your tablets?

(Check all that apply)

__ Partner

__ Spouse

__ Family member

__ Friend

__ Neighbor

__ Other, specify: ____________________________________________________

PB14. Estimate how many tablets were used by someone other than you.

__ __ Tablets

97 Don't Know

PB15. Did you obtain any extra supply of pills this month? 1 Yes

0 No Skip to PB18

PB16. Who provided these pills?

(Check all that apply)

__ Clinician

__ Partner

__ Spouse

__ Family member

__ Friend

__ Neighbor

__ Other, specify: ____________________________________________________

PB17. How many extra pills did you receive? __ __ Pills

**SECTION B: Motivation to take PrEP**

PB18. What are your reasons for taking PrEP?

(Check all that apply)

__ PrEP is currently free

__ PrEP is recommended for me by my clinician

__ PrEP will benefit me in the long term

__ My partner has HIV

__ I often have unprotected sex

__ I am afraid of getting HIV

__ Other, specify: ___________________________________________________

PB19. In general, what do you think is your risk of getting HIV from your sex partner(s)?

(Choose one)

1 High risk

2 Moderate risk

3 Low risk

4 No risk

7 Don't Know

PB20. PrEP can lower your chances of getting HIV from your sex partners. Does knowing this increase your motivation to take PrEP?

(Choose one)

1 Yes, very much

2 Yes, somewhat

3 Makes no difference

4 No, not very much

5 No, not at all

PB21. What fears/concerns do you have about taking PrEP?

(Check all that apply)

__ No concerns

__ Taking ARVs makes people think I have AIDS

__ Side effects

__ Taking ARVs makes me afraid

__ I don’t like to take daily pills

__ I prefer to use condoms instead

__ Concerns about stigma

__ Other, specify: ___________________________________________________

**SECTION C: Pill Count (to be completed by staff)**

PB22. Staff initials __ __

PB23. Pills remaining __ __ Pills

PB24. Pills supplied this visit __ __ Pills

PB25. Date of next refill __ __ - __ __ - __ __ __ __ dd - mm - yyyy
