# Supplementary material for: “I wish to remain HIV negative”: Pre-exposure prophylaxis adherence and persistence in transgender women and men who have sex with men in coastal Kenya
Source: PLoS One. 2021 Jan 19;16(1):e0244226. doi: 10.1371/journal.pone.0244226 (PMC7815127; doi:10.1371/journal.pone.0244226)
Supplement: S1 Table — (DOCX) [file pone.0244226.s005.docx]

**S1 Table: Summary of self-reported PrEP adherence among 16 MSM and TGW participants, coastal Kenya**

| IDI  No | Participant | In-depth interview | Days since last PrEP | Days missed taking PrEP | Days in a row missed PrEP | Visual analogue scale | In general, how do you take your PrEP | Pills remaining | TFV levels |
| --- | --- | --- | --- | --- | --- | --- | --- | --- | --- |
| 1 | MSM | Inconsistent | 9 | 6 | 6 | 70 | Most of the time | 3 | BLQ |
| 2 | TGW | Consistent | 1 | 1 | 1 | 96 | All of the time | 28 | LLOQ-349 (<2doses/wk) |
| 3 | MSM | Consistent | 1 | 3 | 3 | 90 | Most of the time | 0 | BLQ |
| 4 | TGW | Consistent | 2 | 0 | 0 | 95 | All of the time | 1 | 700-1249 (4-6doses/wk) |
| 5 | TGW | Inconsistent | 24 | 6 | 6 | 65 | Most of the time | 6 | BLQ |
| 6 | MSM | Inconsistent | 8 | 24 | 24 | 77 | A good bit of the time | 0 | LLOQ-349 (<2doses/wk) |
| 7 | MSM | Consistent | 1 | 0 | 0 | 98 | All of the time | 2 | LLOQ-349 (<2doses/wk) |
| 8 | MSM | Inconsistent | 6 | 6 | 6 | 100 | All of the time | 0 | BLQ |
| 9 | MSM | Inconsistent | 10 | 0 | 0 | 95 | Most of the time | 0 | BLQ |
| 10 | TGW | Consistent | 0 | 6 | 6 | 90 | Most of the time | 6 | 350-699 (2-3doses/wk) |
| 11 | TGW | Consistent | 2 | 3 | 3 | 90 | A good bit of the time | 9 | 700-1249 (4-6doses/wk) |
| 12 | MSM | Inconsistent | 96 | 30 | 30 | 10 | Some of the time | 0 | BLQ |
| 13 | TGW | Consistent | 1 | 0 | 0 | 100 | All of the time | 1 | 700-1249 (4-6doses/wk) |
| 14 | TGW | Consistent | 1 | 0 | 0 | 80 | Most of the time | 0 | BLQ |
| 15 | MSM | Inconsistent | 14 | 14 | 14 | 100 | All of the time | 0 | BLQ |
| 16 | MSM | Inconsistent | 15 | 3 | 3 | 45 | Some of the time | 30 | BLQ |
